# Breast Milk and Allergic Disease Development: Immunological Factors Associated with Eczema, Food Allergy and Wheezing/Asthma

**DOI:** 10.1007/s11882-026-01299-w

**Published:** 2026-10-29

**Authors:** Alessandra Catalogna, Carlo Agostoni, Simona Barni, Roberto Berni Canani, Laura Carucci, Letizia Ciliberti, Serena Coppola, Antonio Corsello, Gregorio Paolo Milani, Francesca Mori, Rita Nocerino, Bernardo Sousa Pinto, Giulia Carla Immacolata Spolidoro, Mattia Giovannini, Daniel Munblit

**Affiliations:** 1 Allergy and Clinical Immunology Unit, Teramo, Italy; 2 Pediatric Unit, Fondazione IRCCS Ca’ Granda Ospedale Maggiore Policlinico, Milan, Italy; 3 University of Milano, Milan, Italy; 4 Allergy Unit, Meyer Children’s Hospital IRCCS, Florence, Italy; 5 Department of Translational Medical Science, University of Naples Federico II, Naples, 80138 Italy; 6 CEINGE Advanced Biotechnologies Research Center, NutriTechLab, University of Naples Federico II, Naples, 80138 Italy; 7 CEINGE Advanced Biotechnologies Research Center, ImmunoNutritionLab, University of Naples Federico II, Naples, 80138 Italy; 8 Task Force for Microbiome Studies, University of Naples Federico II, Naples, 80138 Italy; 9 ImmunoNutritionLab and NutriTechLab at the CEINGE Advanced Biotechnologies Research Center, University of Naples Federico II, Naples, Italy; 10 Department of Health Sciences, University of Florence, Florence, Italy; 11 Department of Clinical-Surgical, Diagnostic and Pediatric Sciences, University of Pavia, Pavia, Italy; 12 Pediatric Gastroenterology Unit, Children’s Hospital “G. Di Cristina”, ARNAS Civico-Di Cristina-Benfratelli, Palermo, Italy; 13 Department of Pharmacy, Health and Nutritional Sciences, Rende-Pediatric Unit, Società Italiana di Medicina di Emergenza e Urgenza Pediatrica (SIMEUP), University of Calabria, Annunziata Hospital, Cosenza, 87100 Italy; 14 Department of Community Medicine, Information and Health Decision Sciences, Faculty of Medicine, MEDCIDS, University of Porto, Porto, Portugal; 15 Centre for Health Technology and Services Research, Health Research Network, Faculty of Medicine, CINTESIS@RISE, University of Porto, Porto, Portugal; 16 Pediatric Rheumatology Unit, Department of Childhood and Adolescent Medicine, ASST Fatebenefratelli-Sacco, Milan, Italy; 17 Care for Long Term Conditions Division, Florence Nightingale Faculty of Nursing, Midwifery and Palliative Care, King’s College London, London, UK; 18 Department of Paediatrics and Paediatric Infectious Diseases, Institute of Child’s Health, Sechenov First Moscow State Medical University (Sechenov University), Moscow, Russia

**Keywords:** Human milk, Food allergy, Eczema, Asthma, Immunomodulation, Transforming growth factor-beta, Secretory IgA, Human milk oligosaccharides

## Abstract

**Purpose of Review:**

Human milk (HM) contains a diverse array of immunological, microbial, and metabolic bioactive factors that may influence early immune maturation and allergic disease susceptibility. This narrative review examines current evidence linking major HM bioactives with eczema, food allergy, and wheezing/asthma, integrating mechanistic findings with human observational and clinical data.

**Recent Findings:**

Transforming growth factor-β (TGF-β), secretory IgA (sIgA), human milk oligosaccharides (HMOs), cytokines, fatty acids, short-chain fatty acids, the HM microbiome, and extracellular vesicles have all been implicated in pathways relevant to epithelial integrity, microbial colonization, and immune tolerance. However, associations with allergic outcomes are heterogeneous and frequently context dependent. Findings vary according to lactation stage, maternal characteristics, infant susceptibility, microbial exposures, and outcome definition. Mechanistic evidence is often stronger than prospective clinical evidence, and no individual HM component or bioactive profile has yet emerged as a reproducible predictor of allergy protection.

**Summary:**

Current evidence supports viewing HM as a dynamic maternal–milk–infant biological network rather than as a source of isolated protective factors. Future studies should integrate repeated HM sampling, rigorous allergic phenotyping, and multi-omic approaches to distinguish causal mediators from biomarkers of maternal or infant biology.

## Introduction

The “neonatal window of opportunity” encompasses a critical prenatal and early postnatal period during which nutritional and environmental exposures shape immune maturation and may influence susceptibility to immune-mediated diseases [1]. Allergic diseases are among the most common chronic conditions in childhood, affecting approximately 10–30% of children worldwide, with food allergy occurring in about 8% [2–4].

Although human milk (HM) provides well-established protection against infections and breastfeeding is recommended for its nutritional and immunological benefits, its role in preventing allergic sensitization and disease remains less clearly defined [5, 6].

HM should not, however, be regarded as a uniform nutritional exposure. Its composition changes substantially across colostrum, transitional milk, and mature milk and varies among mothers [7–10].

In addition to nutrients, HM can contain trace dietary antigens transferred after maternal ingestion, together with cytokines, immunoglobulins, growth factors, human milk oligosaccharides (HMOs), microbial communities, and bioactive fatty acids that may influence mucosal immune maturation, epithelial function, and gut microbial colonization [11–19]. This interindividual and temporal variability provides a biological rationale for examining HM composition, rather than breastfeeding status alone, in relation to allergic outcomes.

Epidemiological findings mirror this complexity. Meta-analyses have reported associations between breastfeeding and lower risks of food allergy, recurrent wheezing/asthma, or eczema, particularly in early childhood, whereas other studies show little or no protective association [20–26]. A Cochrane review found no evidence that exclusive breastfeeding beyond 3–4 months reduced eczema, asthma, or other allergic conditions, while the ISAAC Phase Two study, including more than 51,000 children from 21 countries, found no strong association with eczema risk [27, 28]. Differences in breastfeeding duration and exclusivity, complementary feeding, study populations, outcome definitions, and the frequent treatment of HM as a uniform exposure probably contribute to these inconsistencies. Early introduction of complementary foods, particularly allergenic foods, may further modify allergy risk, emphasizing that breastfeeding operates within the broader context of early-life nutrition [29–31].

HM may be particularly relevant to mucosal immune development during the first months of life, when the gastrointestinal tract, microbiota, and immune system undergo rapid maturation [28, 32]. This interaction may be especially relevant in infants with atopic dermatitis, in whom epithelial barrier dysfunction, food sensitization, and complementary feeding intersect early in the course of allergic disease [33]. These early interactions may contribute to subsequent allergic trajectories, known as “atopic march”, although whether specific HM components modify such progression remains uncertain.

Against this background, this review examines major immunomodulatory and bioactive components of HM and integrates mechanistic and human evidence relating them to eczema, food allergy, and wheezing/asthma, the allergic outcomes most extensively investigated in relation to HM composition and early immune programming. Evidence for other allergic outcomes, including allergic rhinitis and anaphylaxis, remains comparatively limited and was therefore not a primary focus. Particular attention is given to sources of heterogeneity across studies and to the distinction between biological plausibility, observational associations, and evidence relevant to clinical prevention.

## Human Milk (HM) as an Immunological Interface in Early Life

HM is a dynamic biological system whose composition changes throughout lactation to accommodate the evolving nutritional and immunological needs of the infant. Colostrum, transitional milk, and mature milk differ quantitatively and qualitatively in their bioactive composition, with colostrum particularly enriched in cytokines, leukocytes, and immunoglobulins [7–9]. Beyond macronutrients, HM contains a broad range of immune-active factors, including cytokines, chemokines, immunoglobulins, HMOs, and viable immune cells [10]. Variation in cellular composition has also been reported in association with cow’s milk allergy, although the clinical relevance of these observations remains incompletely defined [34]. These components may influence allergic disease through several interconnected pathways, including maturation of mucosal immunity, maintenance of epithelial barrier integrity, regulation of inflammatory responses, and establishment of the infant gut microbiota. Cytokines such as transforming growth factor-β (TGF-β), IL-10, IL-1β, IL-6, IL-13, and IFN-γ are particularly abundant during early lactation and participate in immune regulation and tolerance-related pathways [35–37]. Their concentrations, however, vary considerably between mothers and populations and may be influenced by maternal diet, environmental exposures, atopic background, and geographical setting [38]. This variability complicates attempts to relate individual HM components to subsequent allergic outcomes.

The biological effects of HM bioactives also depend on the recipient infant. Features of the immature gastrointestinal tract may favour the persistence and activity of milk-derived immune mediators during early life. The relatively high gastric pH of young infants may limit degradation of some cytokines and immunoglobulins, while soluble receptors and receptor antagonists may further modify cytokine bioavailability and signalling [39]. Thus, measured concentrations in milk do not necessarily translate directly into biological exposure or effect. HM also provides a microbial interface between mother and infant. Milk-associated microbial communities may contribute to infant gut colonization and immune development, although their composition varies between mothers and associations with allergic outcomes remain observational [12, 13].

Additional metabolites and nutritional bioactives, including short-chain fatty acids such as butyrate and PUFAs, may further contribute to epithelial and immune pathways relevant to allergic disease [14, 16, 17]. Their availability in HM is influenced by maternal characteristics and diet, further underscoring the dynamic nature of the maternal–milk–infant interface [15, 16].

**Fig. 1:**
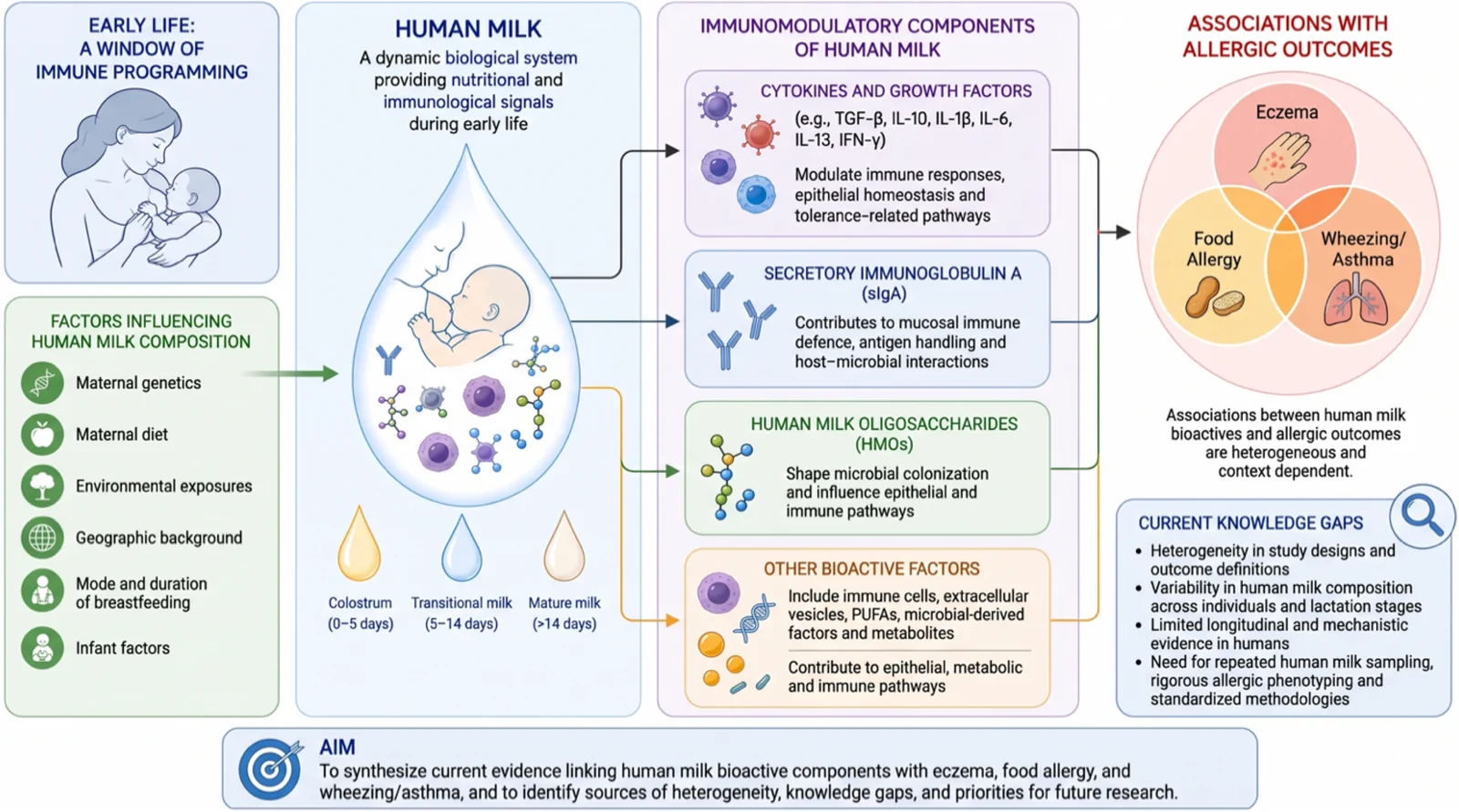
Overview of major human milk bioactive components, factors influencing their composition, and potential pathways linking human milk with allergic outcomes. Created in BioRender. CATALOGNA, A. (2026) https://BioRender.com/8l96k

Collectively, HM should therefore be considered a heterogeneous immunological exposure rather than a uniform biological exposure. Its bioactive components act within an interconnected maternal–milk–infant system involving mucosal immunity, epithelial function, microbial colonization, and immune programming [19]. A schematic overview of the major factors influencing HM composition, its principal bioactive components, and their potential links with allergic outcomes is shown in Fig. 1.

The following sections examine the major components of this system and the strength of the evidence linking them to subsequent allergic outcomes. Key human studies examining associations between HM bioactives and eczema, food allergy, and wheezing/asthma are summarized in Tables 1, 2 and 3, respectively.

**Table 1:** Human milk bioactives and their associations with eczema

ECZEMA									
Author, year	Country	Study design	HM stage	N of participants	Immunological markers assessed	Outcome	Outcome definition	Study findings	time of follow-up
Businco et al., 1993 [107]	Italy	Cross-sectional case-control study	M	23 mothers of infants with atopic eczema; 18 controls	PUFAs	Eczema	Physician-diagnosed atopic dermatitis	HM from mothers of infants with newly developed atopic eczema had lower levels of DGLA, adrenic acid, and DHA and a higher linoleic acid-to-derivatives ratio than control milk (11.78 vs. 9.02; *P* < 0.01).	Cross-sectional
Kalliomaki et al., 1999 [51]	Finland	prospective cohort study	C, M	47 mother-infant pairs	TGF- β1 TGF- β2	Eczema	Clinical criteria: ≥3/4 major features (pruritus, typical morphology/distribution, chronic/recurrent dermatitis, family history of atopy)	Higher colostral TGF-β1 levels were associated with postweaning- rather than preweaning-onset eczema (*P* = 0.0008) and with postweaning-onset eczema versus no eczema (*P* = 0.0056); TGF-β2 was higher in postweaning- versus preweaning-onset eczema (*P* = 0.015). No differences were observed in M.	1 year
Rautava et al., 2002 [60]	Finland	Double-blind, placebo-controlled trial	M	62 mother-infant pairs	TGF- β1 TGF- β2	Eczema	clinical criteria. Chronic relapsing eczema defined as ≥ 3 episodes lasting at least 1 month in the first 2 years.	Maternal probiotic supplementation was associated with higher HM TGF-β2 concentrations (2885 vs. 1340 pg/mL; *P* = 0.018) and lower occurrence of chronic relapsing eczema (RR 0.32, 95% CI 0.12–0.85; *P* = 0.0098); however, no direct association was observed between HM TGF-β2 concentrations and eczema.	2 years
Hoppu et al., 2005 [108]	Finland	Prospective cohort (high-risk infants)	C, M	34 mother–infant pairs	Fatty acid composition	Eczema	Eczema diagnosed during the 1 st year of life	HM consumed by infants who developed eczema had higher stearic acid levels (8.9% vs. 7.1%; *P* = 0.0009), lower EPA levels (0.10% vs. 0.15%; *P* = 0.02), and higher SAFA/PUFA ratio (*P* = 0.05).	1 year
Rigotti, 2006 [53]	Italy	prospective cohort study	C, M	22 mother-infant pairs (13 allergic mothers, 9 controls)	TGF-β1 IL-10	Eczema	Pediatric examination for signs/symptoms of eczema	Mature HM TGF-β1 concentrations were lower in allergic than non-allergic mothers (215 vs. 1059 pg/mL; *P* = 0.015); 6/13 infants of allergic mothers developed eczema versus 0/9 controls, and 4/6 infants with eczema received HM with undetectable TGF-β1. No significant differences were observed for IL-10.	6 months
Oddy et al., 2006 [109]	Australia	Prospective birth cohort (high-risk families)	M	91 mother–infant pairs	n-3 and n-6 PUFAs	Eczema	Doctor-confirmed eczema; ≥3 predefined clinical criteria. Sensitization assessed by SPT.	A higher HM n-6:n-3 fatty acid ratio at 6 weeks and 6 months was associated with non-atopic eczema at 6 months (*P* = 0.001 and *P* = 0.005, respectively), but not with eczema with sensitization. No associations were observed at 5 years.	6 months and 5 years
Böttcher et al., 2008 [61]	Sweden	Double-blind, placebo-controlled trial	C, M	109 mother-infant pairs (54 probiotic, 55 placebo)	TGF-β1, TGF-β2, IL-10, TNF, sCD14, IgA	Eczema	Pruritic, chronic or chronically relapsing non-infectious eczema with typical features and distribution; IgE-associated if accompanied by sensitization	No association was observed between HM immune parameters and eczema during the first 24 months. Higher colostral TGF-β2 levels showed a non-significant trend toward IgE-associated eczema.	2 years
Ismail et al., 2013 [56]	Australia	Prospective study nested within a randomized DBPC trial	M	79 mother-infant pairs	TGF-β1, sCD14, IgA	Eczema	Eczema according to modified UK Working Party criteria; IgE-associated eczema defined as eczema plus positive SPT	HM sCD14, TGF-β1, and total IgA levels at days 7 and 28 were not associated with eczema during the first 12 months of life.	1 year
Orivuori et al., 2014 [54]	Finland, France, Germany, Switzerland	Prospective cohort study (PASTURE)	M	610 mother-infant pairs	sIgA, TGF-β1	Eczema	Physician-diagnosed on clinical criteria and/or a SCORAD score > 0 at 1 year	Higher HM sIgA levels were inversely associated with eczema up to age 2 (P for adjusted trend = 0.005). Estimated sIgA intake was inversely associated with eczema up to ages 2 (aOR 0.74, 95% CI 0.55–0.99) and 4 years (aOR 0.73, 95% CI 0.55–0.96). No consistent association was observed for TGF-β1.	Up to 6 years
Simpson et al., 2016 [58]	Norway	Analysis nested within a randomized placebo-controlled trial	C, M	259 mother-infant pairs	TGF-β1, TGF-β2, TGF-β3, TSLP	Eczema	Pediatrician-diagnosed eczema using UK Working Party diagnostic criteria	HM TSLP and TGF-β1, TGF-β2, and TGF-β3 concentrations were not significantly associated with eczema by age 2 years.	2 years
Jepsen et al., 2016 [70]	Denmark	prospective cohort study	M	223 mother-infant pairs	19 cytokines and chemokines	Eczema	Pediatrician-diagnosed eczema according to Hanifin and Rajka criteria	Higher HM IL-1β (≥ 0.7 pg/mL) was inversely associated with eczema up to age 3 years (aHR 0.41, 95% CI 0.24–0.68; *P* < 0.001). A HM immune mediator pattern characterized by higher IL-1β, IL-17 A and CCL17 and lower CXCL1 and TSLP was also inversely associated with eczema (aHR 0.82, 95% CI 0.68–0.98; *P* = 0.03).	0–3 years
Munblit et al., 2017 [64]	UK, Russia, Italy	prospective cohort study	C, M	398 mother-infant pairs	IL-2, IL-4, IL-5, IL-10, IL-12, IL-13, IFN-γ, TGF-β1, TGF-β2, TGF-β3, HGF	Eczema	parent-reported eczema	Detectable IL-13 in mature HM was inversely associated with parent-reported eczema (OR 0.18, 95% CI 0.04–0.92), whereas higher TGF-β2 concentrations in mature HM were positively associated with eczema (OR 1.04, 95% CI 1.01–1.06 per ng/mL).	6 and 12 months
Snijders et al., 2006 [59]	Netherlands	Prospective cohort study	M	315 mother-infant pairs	TGF-β1, IL-10, IL-12, sCD14	Eczema	parent-reported eczema by ISAAC criteria	HM TGF-β1, sCD14, and IL-12 concentrations were not associated with eczema during the first 2 years of life.	12 months
Kuitunen et al., 2012 [62]	Finland	double-blind, placebo-controlled, prospective trial	C, M	364 colostrum samples, 321 mature milk samples	TGF-β2, IL-10, total IgA, IgA to cow’s milk, casein, β-lactoglobulin, ovalbumin	Eczema	UK Working Party criteria and considered atopic if IgE-associated	Higher OVA-specific IgA in mature HM was inversely associated with eczema at 2 years (OR 0.55, 95% CI 0.32–0.93; *P* = 0.024), whereas higher TGF-β2 was positively associated with eczema (OR 2.30, 95% CI 1.34–3.94; *P* = 0.003). Colostral TGF-β2 and HM IL-10 were not associated with eczema.	2 years and 5 years
Savilahti et al., 2015 [71]	Finland	Prospective study nested within a randomized, DBPC trial	C, M	260 mother-infant pairs	sCD14, α e β-defensin 2	Eczema	UK Working Party’s criteria	HM sCD14, HNP1–3, and HBD2 levels were not significantly associated with eczema.	5 years
Sprenger et al., 2017 [98]	Finland	Retrospective analysis of the placebo group of a randomized controlled trial	C, M	266 mother-infant pairs	FUT2-dependent HMOs	Eczema	Doctor-diagnosed eczema; IgE-associated eczema defined as eczema plus sensitization	Higher early HM 2′FL levels were associated with lower occurrence of eczema and IgE-associated eczema at 2 years specifically in C-section-born infants (interaction *P* = 0.005 and *P* = 0.003, respectively); no association was observed at 5 years.	2 and 5 years
Morita et al., 2018 [57]	Japan	Nested case-control study within a birth cohort	C, M	96 mother-infant pairs	TGF-β1 and TGF-β2	Eczema	Parent-reported itchy skin rash lasting > 2 months by age 6 months	Higher TGF-β1 in C and lower TGF-β1 in mature HM were observed in infants with eczema. A higher TGF-β1 1-month/C ratio was inversely associated with eczema (middle vs. low: aOR 0.161, 95% CI 0.049–0.534; high vs. low: aOR 0.035, 95% CI 0.006–0.188). No association was observed for TGF-β2.	6 months
Jones et al., 2002 [72]	UK	Prospective birth cohort study	M	29 mother–infant pairs (8 eczema; 21 no eczema)	sCD14	Eczema	Clinical criteria	Lower HM sCD14 levels at 3 months were associated with eczema at 6 months, irrespective of sensitization (*P* = 0.003).	6 months
Logan et al.; 2019 [73]	Germany	Two prospective population-based birth cohorts (UBCS and SPATZ)	M	UBCS: 659; SPATZ: 489	sCD14	Eczema	Clinical criteria	No significant association between sCD14 concentration HM and risk of eczema	3 years
Böttcher et al., 2003 [55]	Sweden	Prospective birth cohort study	C, M	53 mother-infant pairs	IL-4, IL-5, IL-6, IL-8, IL-10, IL-13, IL-16, IFN-γ, TGF-β1, TGF-β2, RANTES, eotaxin, sIgA	Eczema	Clinical criteria	No significant association between HM cytokines, chemokines or sIgA and allergic symptoms, including eczema, during the first 2 years of life.	2 years
Lodge et al., 2021 [99]	Australia	Prospective birth cohort study	C, M	285 mother-infant pairs	HMOs	Eczema	Clinical criteria	“Acidic Lewis” HMO profile was associated with higher odds of eczema at 6 months (OR 4.24, 95% CI 1.36–13.24; *P* = 0.013); a similar but non-significant association was observed at 12 years (OR 3.68, 95% CI 0.95–14.27; *P* = 0.06).	12 years
Tomic̆ić et al., 2010 [52]	Sweden Estonia	Prospective cohort study	C, M	99 (39 Estonian, 60 Swedish mother-infant pairs)	TGF-β1, TGF-β2, SIgA, IL-4, IL-10, IL-13, IFN-γ	Eczema	Physician-diagnosed eczema – clinical criteria	No significant association between HM IL-4, IL-10, IL-13, IFN-γ, TGF-β1, TGF-β2 or SIgA levels and eczema up to 24 months.	2 years
Ochiai et al., 2013 [36]	Japan	Nested case-control study within a prospective birth cohort	C, M	98: 49 infants with AD, 49 healthy controls	26 cytokines/chemokines	Eczema	Itchy eczema lasting ≥ 2 months at 6 months of age, assessed via parental questionnaires.	Higher IL-1β and IL-12p40 in C, and IL-4, eotaxin, G-CSF, GM-CSF, IFN-α2 and MIP-1α in mature HM, were observed in infants who developed eczema. High mature-HM eotaxin remained associated with eczema in adjusted analysis (OR 3.30, 95% CI 1.16–9.34).	6 months

**Table 2:** Human milk bioactives and their associations with food allergy

FOOD ALLERGY									
Author, year	Country	Study design	HM stage	N of participants	Immunological markers assessed	Outcome*	Outcome definition	Study findings	time of follow-up
Kalliomaki et al., 1999 [51]	Finland	prospective cohort study	C, M	47 mother-infant pairs	TGF- β1 TGF- β2	FA (CMA)	Clinical symptoms resolving after cow’s milk elimination and recurring after DBPC cow’s milk challenge	Higher colostrum TGF-β2 levels were associated with food antigen-specific IgA responses at 3 months (*P* = 0.048); a similar trend was observed for TGF-β1 (*P* = 0.076).	12 months
Saarinen et al., 1999 [67]	Finland	prospective cohort study	C	6209 infants; 118 developed CMA; C available for 108 CMA cases	TGF-β1, IL-6, IFN-γ, total IgA and IgM, CM-specific IgA antibodies	FA (CMA)	Typical symptoms resolving after CM elimination and recurring during hospital-based CM challenge	C TGF-β1 was lower in infants who developed IgE-mediated than non-IgE-mediated CMA (589 vs. 1162 pg/mL; *P* = 0.012) but did not differ significantly between CMA cases and controls. Higher TGF-β1 was associated with greater IgA/IgG responses and lower skin-test and cellular reactivity to CM proteins.	6.4 months (median age at challenge test), up to 18 months
Rautava et al., 2002 [60]	Finland	Double-blind, placebo-controlled randomized trial	M	62 mother-infant pairs	TGF- β2	FA (CMA)	DBPC challenge when symptoms, clinical signs, or SPT suggested CMA	Maternal probiotic supplementation increased HM TGF-β2 (2885 vs. 1340 pg/mL; *P* = 0.018), but HM TGF-β2 was not directly associated with atopic disease or sensitization. CMA frequency did not differ significantly between probiotic and placebo groups (21% vs. 10%).	2 years
Munblit et al., 2017 [64]	UK, Russia, Italy	Prospective multicentre cohort study	C, M	398 mother-infant pairs	IL-2, IL-4, IL-5, IL-10, IFNγ, IL-12, IL-13, HGF, TGFβ−1, −2, −3	Parent reported FA	parent-reported FA/sensitivity/intolerance	Detectable IL-13 in C was associated with lower odds of parent-reported FA at 6 months (OR 0.10, 95% CI 0.01–0.83).	12 months
Snijders et al., 2006 [59]	Netherlands	Prospective birth cohort study	M	315 mother-infant pairs	TGF-β1, IL-10, IL-12, sCD14	Food sensitization only	Serum specific IgE > 0.3 IU/mL to ≥ 1 tested food or inhalant allergen at 2 years	No association between HM TGF-β1, sCD14 or IL-12 concentrations and allergic sensitization at 2 years. IL-10 was undetectable.	12–24 months
Joseph et al., 2014 [65]	USA	Prospective cohort study	M	304 mother-infant pairs	TGF-β1	FA	FA expert physician panel-classified; not OFC-confirmed	No overall association with FA; a non-significant inverse association was observed among non-Black infants of non-atopic mothers (aOR 0.08, 95% CI 0.004–1.51).	2–3 years
Berdi et al., 2019 [66]	France	Prospective cohort study	C, M	318 (72 FA cases)	50 immune/growth factors	Parent-reported FA	Parental report of a physician diagnosis of FA during the first 5 years; no food-specific IgE or food challenge performed	Higher CXCL10, TNF-β and IL-2 concentrations were associated with higher risk of parent-reported doctor-diagnosed FA after FDR correction (HR 1.02 [95% CI 1.01–1.04], 1.13 [1.06–1.21], and 1.06 [1.03–1.09], respectively).	5 years
Järvinen et al., 2015 [84]	USA	Prospective birth cohort study	M	74 mother–infant pairs (39 non-CMA, 35 CMA)	28 immune components; TGF-β; BLG- and casein-specific IgA;	FA (CMA)	CMA verified by oral food challenge, performed at a median age of 5.5 months (IQR 3.1–7.7)	Lower IL-1β, IL-10 and BLG-specific IgA levels were associated with CMA after adjustment for maternal covariates (*P* = 0.001, 0.04 and 0.005, respectively); lower IL-6 showed a non-significant trend (*P* = 0.07).	Median 5.5 - up to 7.7 months
Miliku et al., 2018 [96]	Canada	Prospective birth cohort study (CHILD)	M	421 mother-infant pairs	HMOs	Food sensitization only	Food sensitization at 1 year assessed by standardized skin prick testing to food allergens	No individual HMO or total HMO concentration was associated with food sensitization; however, overall HMO profiles differed between sensitized and non-sensitized infants (*P* < 0.001; AUC 0.73, 95% CI 0.66–0.79). The lowest vs. highest PLS-DA score quintile was associated with lower odds of food sensitization (aOR 0.10, 95% CI 0.03–0.34).	1 year
Lodge et al., 2021 [99]	Australia	Prospective birth cohort study (MACS)	C, M	285 mother-infant pairs	HMOs	Food sensitization only	Positive SPT to ≥ 1 food allergen	The acidic-predominant HMO profile (high 6′SL and LSTc) was associated with lower odds of food sensitization at 2 years (OR 0.11, 95% CI 0.01–0.97) and 12 years (OR 0.09, 95% CI 0.01–0.69), compared with the neutral Lewis profile.	18 years
Järvinen et al., 2014 [80]	Finland	Prospective birth cohort study	M	145 mother-infant pairs	Casein- and BLG-specific IgA and IgG; total IgA	FA (CMA)	OFC at median 5.8 months	Lower BM casein- and BLG-specific IgA levels were associated with delayed-type CMA (*P* = 0.007 and *P* = 0.008, respectively).	Median 5.8- up to 18 months
Seppo et al., 2017 [90]	USA, Finland	Prospective birth cohort study	M	80 (41 non-CMA, 39 CMA)	HMOs	FA (CMA)	OFC at a median of 6 months	Lower BM LNFP III concentrations were associated with CMA after correction for multiple comparisons (29 vs. 57 µM; adjusted *P* = 0.0069); infants receiving milk with LNFP III < 60 µM had higher odds of CMA (OR 6.7, 95% CI 2.0–22).	18 months
Järvinen et al., 2000 [81]	Finland	Prospective birth cohort study	C, M	87 mother-infant pairs (48 CMA, 8 disease controls, 31 controls)	Total IgA, Cow’s milk-specific IgA	FA (CMA)	symptoms + OFC at a median of 4.9 months	Lower total HM IgA was associated with CMA; total IgA < 0.25 g/L between 6 days and 4 weeks postpartum was associated with higher odds of CMA (OR 14.7, 95% CI 3.1–70.2). Cow’s milk-specific IgA was not associated with CMA.	1 year
Lupinek et al., 2019 [87]	Sweden	Prospective birth cohort study (ALADDIN)	M	99 mother-infant pairs	allergen-specific IgG	Food sensitization only	Clinical criteria and/or IgE sensitization	HM allergen-specific IgG profiles correlated with maternal plasma and cord blood IgG; higher maternal allergen-specific IgG was associated with absence of corresponding IgE sensitization at 5 years.	5 years
Paparo et al., 2021 [104]	Italy	Translational experimental study (HM analysis + animal and in vitro models)	C, M	109 healthy lactating women	Butyrate	Mechanistic/translational FA evidence	No clinical FA outcome assessed; experimental allergic responses and tolerogenic mechanisms evaluated in animal and in vitro models.	At concentrations detected in mature HM (median 0.75 mM), butyrate enhanced tolerogenic mechanisms and reduced allergic responses in experimental FA models; clinical prevention of FA in infants was not assessed.	Not available
Wang et al., 2021 [105]	China	Case-control microbiome study	C, M	33 mother–infant pairs	HM microbiota	FA	Symptoms improving with food avoidance and recurring after reintroduction, plus ≥ 1 positive allergy test (allergen-specific IgE, food challenge, or SPT)	HM microbiota composition differed between FA and non-allergic infants; FA was associated with higher abundance of Acinetobacter and Pseudomonas and lower abundance of several genera, including Bifidobacterium and butyrate-producing taxa.	12 months

* Food sensitization was not considered equivalent to clinically confirmed food allergy

**Table 3:** Human milk bioactives and their associations with wheezing/asthma

WHEEZING/ASTHMA									
Author, year	Country	Study design	HM stage	N of participants	Immunological markers assessed	Outcome*	Outcome definition	Study findings	time of follow-up
Jepsen et al., 2016 [70]	Denmark	Prospective cohort study	M	223 mother-infant pairs	19 cytokines and chemokines	Recurrent wheezing	Recurrent wheezing episodes (≥ 2 episodes)	*P* > 0.05 for all comparisons	3 years
Munblit et al., 2017 [64]	UK, Russia, Italy	Prospective cohort study	C, M	398 mother-infant pairs	IL-2, IL-4, IL-5, IL-10, IFNγ, IL-12, IL-13, HGF, TGFβ−1, −2, −3	Recurrent wheezing	Parent-reported wheezing episodes occurring more than once	TGF-β2 and IL-13 levels showed no significant effect on wheezing risk (*P* > 0.05)	1 year
Orivuori et al., 2014 [54]	Finland, France, Germany, Switzerland, Austria	Prospective cohort study (PASTURE)	M	610 mother-infant pairs	sIgA, TGF-β1	Asthma	Doctor’s diagnosis of asthma or wheezy bronchitis	*P* > 0.05	4 years and 6 years
Snijders et al., 2006 [59]	Netherlands	Prospective cohort study	M	315 mother-infant pairs	TGF-β1, IL-10, IL-12, sCD14	Wheezing	at least one wheezing attack reported by parents in the 7-, 12-, or 24-month postpartum questionnaires	*P* > 0.05	24 months
Savilahti et al., 2005 [68]	Finland	Prospective cohort study	C	228 mother-infant pairs	IgA antibodies to cow’s milk (CM), casein, sCD14, TGF-β1, TGF-β2	Asthma	at least 3 episodes of bronchial obstruction responsive to a bronchodilator + confirmation by a paediatrician.	No significant association between IgA, TGF-β1, TGF-β2, or sCD14 and asthma risk; *P* > 0.05 for all comparisons	4 years
Soto-Ramírez et al., 2012 [69]	USA	Longitudinal cohort study	M	115 mother-infant pairs	IFN-γ, CXCL10, IL-6, CXCL8, IL-5, IL-13, TGF-β1, IgA	Asthma-like symptoms	ISAAC questionnaire at 6 and 12 months	Higher HM IL-5 and IL-13 were associated with higher risk of asthma-like symptoms, whereas higher HM IgA and TGF-β1 were associated with lower risk.	6 months and 12 months
Böttcher et al., 2003 [55]	Sweden	Prospective birth cohort study	C, M	53 mother-infant pairs	IL-4, IL-5, IL-6, IL-8, IL-10, IL-13, IL-16, IFN-γ, TGF-β1/β2, RANTES, eotaxin, sIgA	Asthma	3 or more episodes of bronchial obstruction, at least once verified by a physician	P-values were not significant	2 years
Lodge et al., 2021 [99]	Australia	Prospective birth cohort study	C, M	285 mother-infant pairs	HMOs	Wheezing/Asthma	Questionnaire-reported wheeze; asthma defined by reported episodes and/or asthma medication use.	The “acidic Lewis” HMO profile was associated with higher odds of wheeze at 12 years (OR 3.80, 95% CI 1.06–13.60; *P* = 0.040) and 18 years (OR 6.10, 95% CI 1.57–23.74; *P* = 0.009).	18 years
Wijga et al., 2006 [110]	The Netherlands	Prospective birth cohort (PIAMA study)	M	265 mother–infant pairs	n-3, n-6 and trans fatty acids	Asthma	Doctor-diagnosed asthma with active symptoms and/or asthma medication use	In children of mothers with allergy, higher HM DHA and n-3LCP/n-6LCP ratio were inversely associated with asthma at 4 years (OR 0.39 for both).	4 years
Soto-Ramírez et al., 2012 [111]	USA	Prospective longitudinal cohort study	C, M	231 pregnant women recruited; 78 M samples (24 C samples)	n-3 and n-6 PUFAs	Asthma-like symptoms	Recurrent asthma-like symptoms (wheezing, wheezing attacks, noisy breathing or shortness of breath) assessed by ISAAC questionnaire	High total n-6 PUFAs in mature HM were associated with asthma-like symptoms (adjusted RR 3.11, 95% CI 1.35–7.19 after adjustment for total n-3 PUFAs); no significant association was observed for total n-3 PUFAs.	6 and 12 months

* Early-life wheezing and asthma were retained as distinct outcomes because infant wheezing is not equivalent to persistent childhood asthma

*HM* Human Milk, *C* Colostrum, *M* Mature Milk, *TSLP* Thymic Stromal Lymphopoietin, *HGF* Hepatocyte Growth Factor, *HNP* Human Neutrophil Peptide, *HBD2* Human Beta-Defensin 2, *CXCL1* C-X-C Motif Chemokine Ligand 1, *CXCL8* C-X-C Motif Chemokine Ligand 8, *CXCL10* C-X-C Motif Chemokine Ligand 10, *CCL17* C-C Motif Chemokine Ligand 17, *G-CSF* Granulocyte Colony-Stimulating Factor, *GM-CSF* Granulocyte-Macrophage Colony-Stimulating Factor, *MIP-1α* Macrophage Inflammatory Protein 1 Alpha, *RANTES* Regulated Upon Activation, Normal T Cell Expressed and Presumably Secreted, *HMO* Human Milk Oligosaccharide, *FUT2* Fucosyltransferase 2, *LNFP III* Lacto-N-Fucopentaose III, *6′SL* 6′-Sialyllactose, *LSTc* Sialyllacto-N-Tetraose c, *PUFA* Polyunsaturated Fatty Acid, *DHA* Docosahexaenoic Acid, *EPA* Eicosapentaenoic Acid, *DGLA* Dihomo-γ-linolenic Acid, *SAFA* Saturated Fatty Acid, *OVA* Ovalbumin, *BLG* Beta-Lactoglobulin, *CM* Cow’s Milk, *FA* Food Allergy, *CMA* Cow’s Milk Allergy, *SPT* Skin Prick Test, *SCORAD* SCORing Atopic Dermatitis, *OFC* Oral Food Challenge, *DBPC* Double-Blind Placebo-Controlled, *OR* Odds Ratio, *aOR* Adjusted Odds Ratio, *HR* Hazard Ratio, *aHR* Adjusted Hazard Ratio, *RR* Relative Risk, *AUC* Area Under the Curve, *PLS-DA* Partial Least Squares Discriminant Analysis, *FDR* False Discovery Rate, *IQR* Interquartile Range, *n-3LCP* n-3 Long-Chain Polyunsaturated Fatty Acids, *n-6LCP* n-6 Long-Chain Polyunsaturated Fatty Acids

### Cytokines and Soluble Immune Mediators

Cytokines and soluble immune mediators in HM provide a potential pathway through which maternal immune signals may influence immune maturation during early life. Among these factors, TGF-β has been the most extensively investigated in relation to allergic disease. TGF-β is abundant in HM and exists as three isoforms, with TGF-β2 predominating over TGF-β1; concentrations of both isoforms generally decline as lactation progresses [40, 41]. Its biological effects include regulation of inflammatory responses and promotion of immune tolerance, providing a strong mechanistic rationale for a potential role in allergy development [42, 43]. TGF-β acts at several levels of the developing mucosal immune system. Experimental evidence indicates that it can inhibit differentiation of naïve T cells toward both Th1 and Th2 phenotypes and promote regulatory T-cell responses [43–45]. It also contributes to intestinal homeostasis by attenuating inflammation, supporting mucosal repair, and influencing gut microbial composition [46, 47]. TGF-β1 has been shown to enhance expression of tight-junction proteins, including zonula occludens-1 and occludin, thereby reinforcing epithelial barrier integrity and potentially limiting transepithelial antigen exposure [48, 49]. However, these effects are context-dependent. In the presence of IL-6, TGF-β may favor Th17 differentiation, whereas retinoic acid produced by intestinal CD103 + dendritic cells can shift this balance toward regulatory T-cell responses [50]. This pleiotropic biology may partly explain why epidemiological studies have not identified a consistent relationship between HM TGF-β concentrations and allergic disease.

Indeed, clinical findings for eczema are heterogeneous. Associations with both lower and higher risk have been reported for TGF-β1 and TGF-β2, while several prospective cohorts and intervention studies found no significant relationship [51–64]. Some findings also suggest that the timing and relative change in TGF-β concentrations across lactation may be more informative than a single measurement. Maternal allergic status may further influence HM TGF-β1 concentrations, as lower TGF-β1 levels have been reported in milk from allergic mothers. Overall, however, current evidence does not support a consistent protective association between either TGF-β isoform and eczema, in agreement with previous systematic evidence [42]. A similarly heterogeneous picture emerges for food allergy. Higher TGF-β concentrations have been associated with enhanced tolerance-related immune responses, including IgA responses in some cohorts, and lower TGF-β1 concentrations have been reported in association with IgE-mediated cow’s milk allergy or increased food allergy risk in selected populations [51, 65–67]. Conversely, other studies, including cohorts assessing clinically defined food allergy, found no significant association with TGF-β1 or TGF-β2 [59, 60]. Interpretation is further complicated by differences in outcome ascertainment, ranging from sensitization or parent-reported reactions to oral food challenge-confirmed allergy. Thus, although the effects of TGF-β on regulatory immunity and epithelial homeostasis provide biological plausibility, human data do not establish a consistent protective association between HM TGF-β and food allergy. Evidence for respiratory outcomes is also inconsistent. Higher HM TGF-β1 concentrations were associated with a lower risk of asthma-like symptoms during infancy in one cohort, whereas other studies reported no significant associations between TGF-β1 or TGF-β2 and wheezing or physician-diagnosed asthma [54–56, 59, 64, 68, 69]. These discrepancies are particularly difficult to interpret because early-life wheezing encompasses heterogeneous phenotypes and is not equivalent to persistent childhood asthma. Overall, the available evidence suggests that any relationship between HM TGF-β and allergic disease is likely to depend on isoform, concentration, timing of exposure, maternal characteristics, and the clinical phenotype assessed rather than reflecting a uniform protective effect.

Other HM cytokines have been studied less extensively but further illustrate this context dependency. In a prospective cohort, high HM IL-1β concentrations were associated with a lower risk of eczema through 3 years of age [70]. Detectable IL-13 in mature milk was similarly associated with a lower risk of eczema, and detectable colostrum IL-13 with a lower risk of parent-reported food allergy in one multicountry cohort [64]. In contrast, higher HM IL-5 and IL-13 concentrations were associated with an increased risk of asthma-like symptoms during infancy in another longitudinal cohort [69]. Other studies examining IL-4, IL-5, IL-10, IFN-γ, and related cytokines found no significant associations with eczema or respiratory outcomes. [52, 53, 55, 59, 64, 70] These findings argue against assigning individual cytokines an intrinsically protective or risk-enhancing role across allergic phenotypes.

Limited evidence also links cytokine profiles with food allergy. Lower HM concentrations of IL-1β, IL-6, and IL-10 were observed among infants with cow’s milk allergy in one prospective cohort,100 while another large cohort identified associations between selected mediators, including CXCL10, TNF-β, and IL-2, and subsequent food allergy risk [66]. These observations remain exploratory, however, and have not been consistently replicated across populations. Across allergic phenotypes, increased eotaxin concentrations in mature milk were associated with subsequent atopic dermatitis in one study, whereas broader cytokine panels have frequently yielded predominantly null associations [36, 52, 55, 59]. Soluble CD14 (sCD14), an innate immune mediator involved in microbial recognition, has also produced conflicting findings. Higher HM sCD14 concentrations have been associated with eczema in some cohorts, whereas lower concentrations were associated with increased eczema risk in others, and several studies reported no association [56, 59, 71–73].

Lower colostrum sCD14 was associated with increased risk of atopy in one study that also assessed food allergy-related outcomes, while no consistent relationship has emerged for wheezing or asthma [56, 59, 68].

TSLP has likewise been investigated in the context of eczema, but available intervention data did not support a mediating role for HM TSLP in the observed effects of maternal probiotic supplementation [58].

Collectively, cytokines and soluble immune mediators provide compelling mechanistic links between HM exposure, epithelial homeostasis, and early immune programming, but their associations with allergic outcomes remain inconsistent. TGF-β is the most extensively studied example: despite multiple tolerogenic mechanisms, epidemiological findings include inverse, positive, and null associations. Similar outcome- and context-dependent patterns are observed for IL-13, IL-1β, sCD14, and other mediators. Differences in milk sampling, lactation stage, assay methodology, maternal atopic status, and definitions of allergic outcomes likely contribute substantially to this variability [42, 52, 74].

Current evidence therefore supports a role for HM cytokines as components of a complex immunological network rather than as isolated biomarkers or determinants of subsequent allergic disease.

### Secretory Immunoglobulins (sIgA) and Antigen-Specific Immunity

Secretory immunoglobulin A (sIgA) is the predominant immunoglobulin in HM and represents an important component of mucosal immune protection during a period in which endogenous intestinal IgA production remains immature [75–79]. Concentrations are highest in colostrum, typically ranging from 5 to 10 g/L, and decline during lactation to approximately 0.5–1 g/L in mature milk. Unlike circulating IgG, sIgA acts predominantly at mucosal surfaces, where it contributes to epithelial defence, coats commensal bacteria, and limits interactions between luminal antigens and the developing immune system. These functions provide a biologically plausible link between HM immunoglobulins, microbial colonization, and the establishment of immune tolerance. Consistent with this mucosal role, breastfed infants have been reported to develop higher fecal sIgA concentrations than formula-fed infants during the first weeks of life, suggesting that HM feeding may support early gastrointestinal IgA responses [78].

HM sIgA is also part of a dynamic maternal–infant immune axis. Its concentration and antigen specificity are influenced by maternal antigen exposure, diet, and immune status [80, 81]. IgA-producing plasma cells originating from the maternal gut migrate to the mammary gland through the enteromammary pathway, allowing milk antibodies to reflect maternal exposure to dietary and environmental antigens. Oral immunization during lactation has been shown to increase IgA-secreting cells in HM without comparable increases in serum or saliva, further supporting a compartmentalized gut–mammary immune pathway [82, 83]. Consistent with this compartmentalization, food-specific IgA in HM shows epitope-recognition patterns that differ from paired maternal serum, indicating that the milk antibody repertoire is not simply a reflection of systemic maternal IgA [82].

Maternal dietary proteins may also be transferred into HM in small and highly variable amounts; a recent human study detected peanut, egg, and cow’s-milk proteins after maternal ingestion, with milk-derived Ara h 2 retaining IgE-crosslinking capacity [11]. Whether such low-dose antigen exposure promotes tolerance, sensitization, or neither in breastfed infants remains unresolved. Thus, total sIgA concentration alone may not fully capture the biological information transferred through HM; antigen specificity and the timing of exposure may be equally relevant.

Among allergic outcomes, evidence for eczema suggests a possible inverse association with HM sIgA, although findings are not uniform. In the PASTURE cohort, higher sIgA exposure during the first year of life was associated with lower odds of atopic dermatitis at 2 and 4 years [54].

In contrast, other studies assessing secretory or total HM IgA reported no significant association with eczema or broader allergic outcomes during infancy [55, 56]. Antigen-specific responses add further complexity: lower ovalbumin-specific IgA in mature milk was associated with increased eczema risk in one study, whereas other investigations of total IgA or broader immune profiles were null [62]. These findings suggest that associations may depend on milk-sampling timing and on whether total or antigen-specific IgA is measured, rather than reflecting a uniform relationship between HM IgA and eczema.

Among food-allergy outcomes, the most specific evidence concerns cow’s milk allergy (CMA). Lower total HM IgA concentrations in early lactation were associated with a higher likelihood of CMA, with one cohort reporting a markedly increased risk when total IgA concentrations were below 0.25 g/L between 6 days and 4 weeks postpartum [81]. Lower casein- and β-lactoglobulin-specific IgA concentrations have also been reported in mothers of infants who subsequently developed CMA [80, 84].

These findings support the possibility that both the quantity and antigen specificity of HM IgA contribute to mucosal handling of dietary antigens. However, the relationship is not uniformly protective. In a separate study, higher ovalbumin-specific IgA in colostrum was associated with a higher risk of atopy, including eczema and allergic sensitization, by 2 years of age [62]. This apparent discrepancy indicates that allergen-specific IgA should not be interpreted simply as a marker of tolerance; its significance may depend on the antigen involved, lactation stage, maternal exposure, and the clinical outcome assessed.

Maternal diet may be one determinant of these antigen-specific responses. A cow’s milk-free maternal diet has been associated with lower cow’s milk-specific IgA concentrations in HM, whereas higher total and milk-protein-specific IgA levels have been associated with a lower risk of CMA in some cohorts [80, 81]. These observations are consistent with the concept that maternal dietary antigen exposure can shape the antibody repertoire transferred through HM. They do not, however, establish that modifying maternal diet alters infant allergy risk, and observational associations between milk antibody profiles and subsequent disease should not be interpreted as evidence for dietary intervention. Consistent with this distinction, current EAACI guidelines suggest against maternal restriction of potential food allergens during breastfeeding for the primary prevention of food allergy in infants and young children [85]. This preventive context should be distinguished from the management of symptomatic breastfed infants, in whom a short, targeted maternal elimination diet may be considered in selected cases when food allergy is clinically suspected [86].

Evidence for respiratory outcomes is limited and inconsistent. Higher HM IgA concentrations were associated with a lower risk of asthma-like symptoms during infancy in one longitudinal cohort, whereas other studies found no significant association between HM IgA and wheezing or physician-diagnosed asthma. [69] Accordingly, the available evidence does not support a reproducible association between HM IgA exposure and later asthma. Interpretation is further limited by heterogeneity in respiratory outcome definitions, particularly the frequent use of infant wheezing as a surrogate for subsequent asthma.

Other HM immunoglobulin classes have received considerably less attention. HM allergen-specific IgG has been proposed as another potential component of antigen-specific immune transfer; however, the available cohort evidence did not demonstrate a statistically significant association between HM allergen-specific IgG and subsequent allergic sensitization or clinical allergy [87] Therefore, its relevance to allergy prevention remains uncertain.

Overall, sIgA provides one of the more biologically plausible links between HM composition and early mucosal immune programming, with the most suggestive epidemiological associations reported for eczema and CMA. Nevertheless, current evidence does not support considering either total or antigen-specific IgA as an isolated predictive biomarker of allergic disease. Differences in antigen specificity, maternal exposure, lactation stage, timing of milk sampling, and outcome ascertainment may substantially modify observed associations. The available data therefore support a model in which HM immunoglobulins participate in antigen-specific immune education within a broader mucosal network rather than exerting a uniform protective effect across allergic diseases.

### Human Milk Oligosaccharides (HMOs) and the Milk–Gut Microbiome Axis

HMOs represent one of the most abundant classes of bioactive components in HM, with more than 200 structurally distinct species identified [10, 88].

Their composition varies substantially among mothers and is influenced by maternal genetics, particularly secretor status, as well as environmental factors [88].

Unlike digestible carbohydrates, HMOs largely reach the distal intestine intact, where they act as selective substrates for specific bacterial communities, particularly *Bifidobacterium* species [89–91]. This functional relationship places HMOs at the interface between HM composition, infant gut colonization, and early immune development.

The infant microbiome is itself shaped by multiple perinatal exposures, including mode of delivery, gestational age, antibiotic use, and feeding practices [92]. Breastfed infants commonly show enrichment of *Bifidobacterium* and *Lactobacillus*, whereas different microbial patterns have been reported in formula-fed infants [92, 93]. Microbial colonization may in turn influence immune maturation, although associations between specific taxa and allergic disease should be interpreted cautiously. Altered early microbial profiles have been reported in infants who subsequently develop atopic phenotypes, consistent with the concept that HM-derived substrates and microbes may contribute to a broader milk–gut–immune axis rather than acting through isolated pathways [91, 94].

HMOs may influence this axis through several complementary mechanisms. In addition to their prebiotic effects, they can act as soluble decoy receptors that interfere with pathogen adhesion and may thereby reduce mucosal inflammatory stimuli [95]. Experimental evidence further suggests effects on epithelial and immune-cell responses, including modulation of pro-inflammatory signaling and regulatory T-cell pathways [96]. Certain HMOs may also influence immune programming independently of microbiome composition, including through epigenetic mechanisms, although the long-term clinical relevance of these pathways remains uncertain [97]. Thus, biologically plausible mechanisms extend beyond a simple prebiotic effect, but evidence from mechanistic studies should not be assumed to translate directly into prevention of allergic disease.

The HMO profile may be more informative than total HMO abundance. Maternal fucosyltransferase 2 (*FUT2*) genotype strongly influences the production of several fucosylated HMOs [88, 98]. In a cohort of infants at high hereditary risk of allergy, higher exposure to FUT2-dependent HMOs was associated with a lower risk of IgE-associated eczema at 2 years among infants born by cesarean section, although this association was not evident at 5 years. This interaction with delivery mode illustrates the likely context dependence of HMO effects and argues against assigning a uniformly protective role to individual oligosaccharides. Conversely, in another high-allergy-risk birth cohort, an “acidic Lewis” HMO profile was associated with higher odds of eczema at 6 months, whereas the association at 12 years was weaker and did not reach statistical significance [99].

Evidence relating HMO profiles to food allergy is similarly heterogeneous and requires careful distinction between sensitization and clinically confirmed disease. In the CHILD cohort, specific HMO profiles were associated with food sensitization during infancy, but food allergy itself was not assessed [96]. Likewise, Lodge et al. reported that an “acidic predominant” HMO profile was associated with a lower likelihood of food sensitization at 2 and 12 years [99]. These findings indicate associations with food sensitization but cannot be interpreted as evidence of protection from clinically confirmed food allergy.

More clinically specific evidence is available for cow’s milk allergy (CMA). In a prospective cohort, lower concentrations of lacto-N-fucopentaose III (LNFP III) were associated with increased odds of CMA, while lower concentrations of 6′-sialyllactose and disialyllacto-N-tetraose were also observed in HM from mothers of infants who developed CMA [90]. In the same study, maternal FUT2 secretor status itself was not significantly associated with CMA risk. These findings suggest that individual HMO structures, rather than secretor phenotype alone, may be associated with CMA. However, the relatively small sample sizes and the lack of replication across independent cohorts limit conclusions regarding specific HMO signatures as predictive biomarkers.

Experimental work provides additional mechanistic support. In an ovalbumin-induced food allergy model, HMO supplementation, including approaches involving 2′-fucosyllactose (2′-FL), reduced allergic responses and was associated with increased regulatory T-cell activity and reduced mast-cell activation [100, 101]. Studies of 2′-FL-containing infant formulas have also reported immune profiles with similarities to those observed in breastfed infants [102]. These findings support biological plausibility but do not establish that supplementation with an individual HMO prevents food allergy in humans.

Respiratory outcomes provide an important counterpoint to a simple protective model. In the longitudinal study by Lodge et al., an “acidic Lewis” HMO profile was associated with increased odds of wheezing at 12 and 18 years and with asthma at 18 years [99]. The persistence of associations from lactational exposure to late adolescence is noteworthy but also raises particular concern regarding residual confounding. Maternal genetics, environmental exposures, diet, and other characteristics associated with HMO profiles may track across childhood; therefore, long-term associations cannot be assumed to represent a direct causal effect of early HMO exposure.

The microbial component of HM further increases this complexity. HM contains bacterial communities including *Lactobacillus*,* Staphylococcus*,* Streptococcus*, and *Bifidobacterium*, and recent genomic studies suggest that interindividual variation in this microbiome may partly reflect maternal genetic background. Higher abundance of *Lawsonella* has been associated with childhood atopy, whereas greater microbial diversity was associated with lower allergy risk; maternal loci including *NPR1* and *COTL1* were also linked to variation in milk microbial composition [12, 13]. These observations are intriguing but remain associative and do not yet support microbiome-based risk stratification in clinical practice.

Overall, HMOs and the HM–gut microbiome form an interconnected biological system with several plausible routes to influence immune development, including selective microbial colonization, epithelial barrier modulation, regulatory immune signaling, and potentially epigenetic programming. Nevertheless, current clinical evidence does not identify a single HMO, HMO profile, or microbial signature that consistently predicts lower allergic disease risk. Associations differ by allergic phenotype, age at outcome assessment, maternal genetic background, mode of delivery, and whether studies measure sensitization or clinically confirmed allergy. HMOs and microbial communities should therefore be considered components of a dynamic ecological network rather than isolated determinants of allergic disease risk.

### Emerging Bioactive Mediators

Beyond classical immune mediators and HMOs, HM contains bioactive metabolites, fatty acids, and extracellular vesicles that may contribute to early immune programming. Among these, short-chain fatty acids (SCFAs), particularly butyrate, polyunsaturated fatty acids (PUFAs), and exosome-associated microRNAs have attracted increasing interest because of their potential effects on epithelial barrier function, regulatory immunity, and host–microbiome interactions [14–18, 103, 104]. However, human evidence linking these mediators to subsequent allergic outcomes remains limited and is substantially less developed than for more extensively studied HM components.

Butyrate is a microbially derived SCFA with well-established immunoregulatory properties and is also naturally present in HM [14, 104]. Stinson et al. reported lower butyrate and acetate concentrations in HM from atopic than non-atopic mothers, although allergic outcomes in their infants were not evaluated [15]. These findings indicate variation in HM SCFA composition according to maternal atopic status but do not link milk SCFA exposure to subsequent allergic disease.

Particularly detailed mechanistic evidence for HM butyrate comes from the translational study by Paparo et al., who detected butyrate in mature HM at physiologically relevant concentrations and investigated its effects across epithelial, immune-cell, and murine food allergy models [104]. In human intestinal epithelial cells, butyrate enhanced the expression of tight-junction proteins, mucins, and human β-defensin-3. In peripheral blood mononuclear cells from children with food allergy, butyrate promoted FOXP3, IL-10, and IFN-γ expression through epigenetic mechanisms and increased regulatory T cells, tolerogenic dendritic cells, and M2 macrophage precursors. In murine food allergy models, butyrate exposure attenuated allergic responses, including Th2-related inflammation. These observations provide a biologically coherent mechanistic framework linking HM butyrate with epithelial barrier integrity and tolerogenic immune pathways.

Microbial findings further support this hypothesis but remain associative. In a small study of mother–infant pairs, HM from mothers of infants who remained non-allergic showed greater bacterial diversity and enrichment of taxa with potential butyrate-producing capacity compared with HM from mothers of infants who developed food allergy [105]. This finding suggests a possible connection between the HM microbiome, microbial metabolites, and tolerance-related pathways but does not demonstrate that milk-derived bacterial butyrate production mediates protection from food allergy. The possibility that maternal diet, microbiota, and breastfeeding jointly influence butyrate availability has prompted interest in whether HM SCFA composition might be modifiable through maternal dietary strategies [106]. At present, however, no prospective birth cohort has demonstrated that higher HM butyrate concentrations independently reduce food allergy incidence, and intervention studies showing that increasing HM butyrate prevents allergic disease are lacking [104–106]. Thus, butyrate should currently be regarded as a mechanistically compelling candidate rather than an established predictive biomarker or preventive target.

PUFAs represent another potentially relevant nutritional–immunological interface, as maternal dietary availability is an important determinant of HM fatty-acid composition. The relative availability of n-3 and n-6 PUFAs has been proposed to influence inflammatory and immune pathways relevant to allergic disease [16, 17]. Early observational evidence showed lower HM n-3 PUFA concentrations among infants who subsequently developed atopic sensitization [18].

Clinical associations with eczema have been reported in several cohorts, although they are not uniform. Breast milk from mothers of infants with newly developed atopic eczema was reported to contain lower long-chain n-3 and n-6 PUFA concentrations and altered linoleic acid metabolite ratios. In a high-risk cohort, lower HM n-3 fatty acids were associated with subsequent atopic dermatitis, while another prospective study found that a higher n-6:n-3 ratio and lower n-3 concentrations were associated with non-atopic eczema at 6 months but not with atopic eczema or outcomes at 5 years [107–109]. These differences suggest that associations with HM fatty-acid composition may vary according to eczema phenotype and age at assessment rather than reflect a uniform relationship with atopic dermatitis.

Respiratory findings similarly suggest context-dependent associations. In the PIAMA birth cohort, higher HM long-chain n-3 PUFA concentrations and a higher n-3:n-6 ratio were associated with lower risks of asthma and persistent allergic symptoms at 4 years among children of allergic mothers, whereas no association was observed among children of non-allergic mothers [110]. In another longitudinal cohort, higher total n-6 PUFAs in mature milk were associated with increased asthma-like symptoms during infancy, while higher n-3 concentrations were associated with reduced atopy but not directly with asthma-like symptoms; no significant associations were observed for colostrum fatty acids [111]. These findings highlight both maternal atopic status and lactation stage as potential effect modifiers and caution against interpreting the n-3/n-6 balance as an isolated determinant of respiratory allergic outcome.

In contrast, evidence linking HM PUFA composition to clinically confirmed food allergy remains limited. Although n-3 PUFAs have plausible immunomodulatory effects and have been proposed as relevant to pediatric food allergy, the available HM studies do not currently provide sufficient evidence to establish specific fatty-acid profiles as predictors of food allergy [17]. This distinction is important when extrapolating mechanistic or nutritional evidence to clinical prevention.

Extracellular vesicles represent an even earlier-stage area of investigation. HM contains exosomes carrying proteins, lipids, and microRNAs with potential immunomodulatory activity [103]. These vesicles may interact with intestinal epithelial and immune cells and provide a mechanism for transferring regulatory molecular signals from mother to infant. In principle, exosomal microRNAs could alter gene expression relevant to epithelial function, inflammation, and immune tolerance. However, direct human evidence linking specific HM exosomal microRNAs to eczema, food allergy, or wheezing/asthma is currently insufficient. Their relevance to allergic disease therefore remains primarily mechanistic and hypothesis-generating.

Collectively, these emerging bioactive mediators broaden the concept of HM from a source of nutrients and soluble immune factors to a complex metabolic and molecular signaling system. Butyrate currently has particularly compelling mechanistic support for epithelial and tolerogenic pathways, whereas observational studies of PUFAs suggest associations that vary by allergic phenotype, maternal atopic status, lactation stage, and timing of outcome assessment. Extracellular vesicles and their microRNA cargo remain promising but clinically underexplored. None of these factors can currently be considered an established predictive biomarker or evidence-based target for allergy prevention, underscoring the need for longitudinal studies integrating repeated HM sampling with metabolomic, lipidomic, microbiome, and immune profiling.

## Why Do Studies Disagree? Sources of Heterogeneity and Knowledge Gaps

The heterogeneity of associations between HM bioactives and allergic outcomes likely reflects not only biological complexity but also substantial differences in study design and exposure assessment. HM is intrinsically dynamic: concentrations of cytokines, immunoglobulins, oligosaccharides, fatty acids, and other mediators vary across lactation and among individuals. Consequently, measurements obtained from colostrum, transitional milk, or a single mature-milk sample cannot necessarily be considered equivalent estimates of infant exposure. More importantly, milk concentration is not synonymous with the actual infant dose, which also depends on the volume and frequency of milk intake, breastfeeding exclusivity, and duration of exposure. Some studies have attempted to approximate cumulative exposure by combining milk concentrations with breastfeeding duration, whereas most have relied on concentration measurements alone [112]. Timing of collection, sample handling and storage, analytical platforms, and assay sensitivity may further contribute to between-study variability, particularly for low-abundance immune mediators [42, 52, 74]. Maternal characteristics provide an additional source of heterogeneity. Atopic status, diet, environmental exposures, geography, and genetic background can all influence HM composition [15, 38, 53]. Maternal genetics are particularly relevant to HMO composition through *FUT2* secretor status, while recent evidence also suggests host-genetic influences on the HM microbiome [12, 13, 88, 98]. These determinants may simultaneously relate to offspring allergy susceptibility, making it difficult to separate the contribution of an individual milk component from the broader maternal–infant biological context. Study populations also differ substantially in baseline allergic susceptibility. Several cohorts specifically enrolled infants with a family history of atopy or other high-risk characteristics, whereas others were population based; such enrichment may influence both absolute disease incidence and the magnitude or direction of associations observed between HM bioactives and allergic outcomes [98, 108, 110]. Similarly, differences in breastfeeding exclusivity and duration, complementary feeding, delivery mode, antibiotic exposure, and other early-life factors may modify both the HM–microbiome axis and subsequent allergic risk.

An additional challenge is that HM bioactives are biologically and statistically correlated. Single-analyte models may therefore attribute an association to one component while partly capturing a broader milk signature, whereas studies testing large biomarker panels in relatively small cohorts are particularly vulnerable to multiple-comparison and cohort-specific findings. Analytical approaches that account for covariance among HM constituents may better reflect the network biology of milk than isolated one-analyte–one-outcome models.

Outcome definition is equally important. Eczema has been variably defined using physician diagnosis, validated criteria, parental questionnaires, and measures of atopic sensitization, potentially grouping biologically distinct phenotypes. Food-allergy studies are particularly vulnerable to outcome misclassification because several cohorts assessed allergen-specific IgE sensitization or parent-reported reactions rather than oral food challenge-confirmed allergy. Sensitization and clinical reactivity should therefore not be treated as interchangeable outcomes. For respiratory disease, recurrent wheezing during infancy is likewise not equivalent to persistent childhood asthma; however, many available studies have not adequately distinguished transient viral-associated wheeze from later atopic asthma.

Differences in follow-up further complicate interpretation. Associations detected during infancy may disappear later in childhood, whereas other studies report relationships persisting into adolescence. Such long-term associations are biologically plausible through durable microbial or immune programming but become increasingly vulnerable to residual confounding from environmental, dietary, and host factors accumulated over time. The predominantly observational nature and relatively small sample sizes of many studies further limit causal inference and increase the likelihood of unstable or cohort-specific findings.

This review also has limitations inherent to its narrative design. The literature search was not intended to be exhaustive, and no formal risk-of-bias assessment of individual studies was performed. Study selection and interpretation are therefore necessarily subject to reviewer judgment, while publication bias may have preferentially increased the visibility of positive or exploratory associations in a literature characterized by numerous relatively small observational studies.

Future studies should therefore move beyond single-component, single-time-point analyses. Repeated HM sampling across lactation, standardized collection and analytical procedures, rigorous phenotype definitions, and prospective follow-up are needed. Particularly valuable will be longitudinal studies integrating HM immunology, metabolomics, lipidomics, HMOs, maternal and infant genomics, and milk and gut microbiomes. Analytical strategies that model correlated HM constituents jointly, rather than treating each bioactive as an independent predictor, will also be important. Such approaches may determine whether apparently inconsistent findings reflect methodological noise or genuine biological interactions between maternal factors, HM composition, infant susceptibility, and timing of exposure. The principal methodological challenges and corresponding research priorities are summarized in Table 4.

**Table 4:** Major sources of heterogeneity and future research priorities in studies of hm bioactives and allergic outcomes

Source of heterogeneity	Current limitation	Future priority
HM sampling and lactation stage	Single samples may not represent longitudinal exposure	Repeated standardized sampling
Infant exposure	Milk concentration ≠ actual infant dose	Incorporate intake, exclusivity and duration
Maternal/infant factors	Atopy, genetics, diet, delivery, antibiotics confound associations	Better characterization and interaction analyses
Correlated HM bioactives	One-analyte models ignore network biology; multiple testing	Multivariable/multi-omic network approaches
Eczema	Heterogeneous phenotype definitions	Standardized diagnostic criteria
Food allergy	Sensitization/parent report often used instead of clinical allergy	OFC-confirmed outcomes
Wheezing/asthma	Early wheeze often conflated with later asthma	Long-term phenotype-specific follow-up
Study design	Small, observational cohorts	Adequately powered prospective cohorts

## Conclusions

HM should be regarded as a heterogeneous and dynamic biological system rather than a uniform exposure. Cytokines, immunoglobulins, HMOs, microbial communities, fatty acids, metabolites, and extracellular vesicles engage multiple plausible pathways through which HM may influence epithelial integrity, microbial colonization, and immune maturation. Nevertheless, no individual HM bioactive has yet shown a sufficiently consistent association across populations and allergic phenotypes to be considered an established predictor or determinant of allergic disease.

The available evidence instead supports a network model in which the biological activity and observed associations of HM components depend on their concentration and timing, interactions with other milk constituents, maternal characteristics, infant genetics, microbial exposures, and the allergic phenotype assessed. The conflicting findings observed for extensively studied mediators such as TGF-β, and the context-dependent associations reported for sIgA, HMOs, SCFAs, and PUFAs, reinforce the limitations of studying these factors in isolation.

Future research should prioritize adequately powered longitudinal cohorts with repeated HM sampling, standardized allergic outcome definitions, and integration of multi-omic approaches. Mechanistic studies should complement, rather than substitute for, prospective clinical evidence and should clarify whether identified bioactive signatures are causal mediators, markers of maternal or infant biology, or both. These approaches may help identify reproducible HM profiles associated with immune tolerance and clarify whether such profiles could eventually inform targeted nutritional strategies.

Clinically, breastfeeding should continue to be encouraged for its established nutritional and immunological benefits; however, this recommendation should not be interpreted as evidence that breastfeeding or specific HM components prevent allergic disease. Current evidence is insufficient to recommend manipulating maternal diet specifically to modify HM bioactives or supplementing individual bioactive factors for allergy prevention. Such approaches remain investigational and require confirmation in appropriately designed prospective and interventional studies before clinical translation.

## Key References

- Khaleva E, et al. Transforming growth factor beta in human milk and allergic outcomes in children: A systematic review. Clin Exp Allergy. 2019;49:1201–1213. https://doi.org/10.1111/cea.13409.
  - This systematic review provides a comprehensive synthesis of evidence on HM TGF-β and allergic outcomes, highlighting substantial between-study heterogeneity and the absence of consistent evidence for a protective association.
- Orivuori L, et al. Soluble immunoglobulin A in breast milk is inversely associated with atopic dermatitis at early age: the PASTURE cohort study. Clin Exp Allergy. 2014;44:102–112. https://doi.org/10.1111/cea.12199.
  - This large prospective cohort reported an inverse association between HM sIgA exposure and early atopic dermatitis, providing important human evidence for a potential link between milk-derived mucosal immunity and eczema risk.
- Lodge CJ, et al. Human milk oligosaccharide profiles and allergic disease up to 18 years. J Allergy Clin Immunol. 2021;147:1041–1048. https://doi.org/10.1016/j.jaci.2020.06.027.
  - This prospective birth cohort examined HMO profiles in relation to allergic phenotypes through adolescence. Its long follow-up highlights potentially persistent associations while also illustrating the challenges of residual confounding in studies of early-life HM exposures.
- Paparo L, et al. Butyrate as a bioactive human milk protective component against food allergy. Allergy. 2021;76:1398–1415. https://doi.org/10.1111/all.14625.
  - This translational study provides mechanistic evidence that butyrate at concentrations detected in HM can enhance epithelial barrier function and promote tolerogenic immune pathways relevant to food allergy; clinical prevention of food allergy in infants was not assessed.
- Burris AD, et al. Secretion of Peanut Protein With IgE Crosslinking Capacity in Human Milk. Allergy. 2026;81:861–873. https://doi.org/10.1111/all.70066.
  - This recent human study demonstrated transfer of dietary peanut, egg, and cow’s-milk proteins into HM after maternal ingestion, including biologically active Ara h 2, while leaving the immunological consequences of this low-dose antigen exposure unresolved.

## Data Availability

No datasets were generated or analysed during the current study.
